# Dislocations—Fractures of the Jaw

**Published:** 1892-03

**Authors:** H. S. Prideaux


ARTICLE II.

### DISLOCATIONS—FRACTURES OF THE JAW.

BY MR. H. S. PRIDEAUX.

A Paper read before the Students' Society, Dental Hospital of London.

*Mr. President and Gentlemen :*

The subject of this paper that I am going to read to you to-night on "Dislocations and Fractures of the Jaw," I shall divide into two parts, viz :—

(1.) Dislocations of the jaw.
(2.) Fractures of the jaw.

Dislocation of the jaw is an accident which does not often come under the notice of the dental surgeon, as it is an occurrence which is generally treated by a general practitioner, but there are times when the dental surgeon's skill is called in, as occasionally the jaw is dislocated by the extraction of a tooth, or during the process of taking an impression, and at such times it would be far more credit to the dentist to reduce the dislocation rather than to have to call in a general practitioner.

As a rule, dislocations of the jaw are fairly easy to reduce at the time of the dislocation, but if they are left for some days or even hours, they will be much more difficult to reduce.

There are four forms of dislocation of the jaw, viz :—

(1.) Complete dislocation.
(2.) Incomplete dislocation.
(3.) Bilateral dislocation.
(4.) Unilateral dislocation.

In complete dislocation of the jaw, one or both of the condyles have slipped out of the glenoid fossæ, and rest right in front of the articulating surfaces of the superior maxillæ.

In incomplete dislocation, the condyles have not slipped out altogether from the glenoid fossæ, but rest on the inter-articulating cartilages.

In bilateral dislocation, both condyles are resting on the inter-articular cartilages, or right outside and in front of the articulating eminence.

In unilateral dislocation, only one condyle is resting on the inter-articular cartilage, or right outside and in front of the articulating eminence, and the other condyle is in its normal position.

To diagnose a dislocated jaw is a very simple matter, and if once seen will never be forgotten.

The first thing you will probably notice is that the mouth is widely open, and that the lower jaw is thrown very much forward, which gives the patient a very idiotic appearance. There is also inability to close the mouth, the centre of the jaw may point directly forward, showing that the patient has a bilateral dislocation, or if the centre of the jaw points either to the right or left, you may be pretty sure that there is a unilateral dislocation.

The causes by which the jaw may be dislocated are many, it may be from laughing very heartily, yawning, or in eating by opening the mouth too widely, or from violence (which is generally direct), such as a sudden blow, or by having a tooth extracted.

People who are of a delicate constitution stand a much greater risk of having the jaw dislocated, as the muscles are not nearly so strong as in a healty and well developed person, and also women are much more liable to dislocation than men.

The treatment of dislocations of the jaw are fairly simple, excepting where the jaw has been dislocated for some time, and has become rather rigid on account of the contraction of the muscles caused by the accident.

The first method I shall mention for reducing the dislocation is, place the patient on a firm low stool or chair, then to stand behind the patient, having previously well padded the thumbs by wrapping them in lint to prevent having them bitten, then place them on the lower molar teeth, with the patient's head firmly resting against your body, you must now press downwards with considerable pressure until you can feel that you have the condyles below the articulating surface of the superior maxillæ, then press a little backwards, and in all probability the condyles will slip back into their normal position, and as they slip into the glenoid fossæ the jaws will be brought together with considerable force, and the thumbs would have probably been badly bitten if you had not taken the precaution to pad them well at the beginning of the operation.

If you fail to get sufficient force in this manner to reduce the dislocation, you should get an assistant to stand behind the patient so as to keep the head rigid, then standing in front of the patient, and go through the same operation as when standing behind the patient, you will most likely reduce the dislocation, as you have much more power standing in front.

If you should fail to reduce the dislocation by these methods, you will have to resort to the use of a lever, which should be about a foot in length and half-an-inch square.

Then by placing one end of the lever on the molar teeth of the dislocated side, and by using the molar teeth of the superior maxilla on the opposite side to the dislocation for the fulcrum, raise the other end held in the hand; then, when you think the inferior maxilla is sufficiently depressed, with the other hand press back the chin, and the condyle will slip into its socket.

If the dislocation is bilateral, you will have to go through the same operation on the opposite side of the mouth.

Always after a dislocation the patient should wear a four-tailed bandage, made partly of elastic, so as to allow the jaw to be used, and at the same time to be a support as well.

After a jaw has once been dislocated, it is always liable to occur again, and patients should always be warned to be very careful not to overstrain it.

### FRACTURES OF THE JAWS.

Fractures of the jaws, like all other bones, can be simple and compound.

A simple fracture is where the bone is broken with a clean fracture, without other injury to the bone or surrounding parts.

A complicated fracture is where the bone is broken in more than one place, and other complications, such as external injuries, injury to vessels, nerves, teeth, and the surrounding tissues.

The inferior maxilla is much more liable to fracture than the superior, the fractures being generally caused by direct violence, such as a blow, fall, or the extraction of a tooth, &c.

The body of the jaw is much more the seat of fractures than the neck or rami of the inferior maxilla, as a blow received on the neck or ramus more often dislocates than fractures it.

The commonest place for the inferior maxilla to be fractured is in the region of the canine tooth.

A fracture of the inferior maxilla is generally compound, there being an opening on the lingual surface of the gum, caused by the rough end of the fractured bones projecting through the mucous membrane, which is not very thick, as a great part of the internal surface of the inferior maxilla is subcutaneous.

The symptoms of fractures in the maxillæ are generally fairly easy to diagnose on account of the mobility of the fractured parts by crepitation, and the irregularity in the line and bite of the teeth.

Fractures of the inferior maxilla in men are much more frequent than in women on account of the greater risk of accident in daily work. The proportion is about ten to one.

Fractures of the superior maxilla are far less frequent than the inferior and the line of fracture does not, as a rule, take any definite position, but is generally very irregular.

There is not, as a rule, much hæmorrhage in fracture of the inferior maxilla, but fracture of the superior maxilla the hæmorrhage is much greater, on account of tearing of the mucous membrane of the many plates of bone which go to make it up.

Fractures of the alveolar border very often cause the teeth to loosen, and on no account should they be extracted at the time of injury.

TREATMENT.—If the superior maxilla is badly crushed it is not wise to meddle with it for a little time, so as to allow the inflammation to subside and the parts begin to heal, you will then be able to gradually bring the bones into opposition by carefully moulding the parts with the hand, you may perhaps have to use a splint but it is not always needed.

Be very careful never to remove any loose bits of bone or gum, as the power of the parts to heal and unite properly is very great on account of the good blood supply, if the teeth are very loose they may be held in position by a wire splint.

Even if the case looks almost hopeless it is surprising what a great change takes place in a few days.

One case I remember where a man was struck with great force in the region of the six upper front teeth, which caused a fracture in the line of junction of the two superior maxillæ, and there was a space between the two central incisors which looked very much as if a tooth had been extracted.

A model was taken, and after it had been sawn in two (where the fracture had taken place), the parts were brought together and a splint made to fit, this was placed in the mouth while the parts were pressed together, it was then wired to the teeth and the patient quickly recovered.

Care should be taken never to use the elevator for extracting an upper molar tooth, as you are almost bound to fracture the process in so doing.

For a simple fracture of the inferior maxilla take a piece of gutta-percha, about 6 inches long and 3 inches wide, and cut the narrow ends for about an inch and a half from the ends towards the centre, then place it in warm water until it is fairly soft, and adjust it to the chin making a kind of cup for it to rest in, having set the fracture, proceed to keep the gutta-percha in position by means of a four-tailed bandage which is made by taking a piece of ordinary bandage about 2 inches wide and a yard long, it is then folded into two, a small slit is now cut in it about 2 inches long, and each end is torn down within about 2 inches of the slit, by so doing the four tails are made, you then stand behind the patient and let the chin rest in the slit, next take the two upper tails bringing them backward along the inferior maxilla and fix the ends behind the neck, the other two tails you bring upwards to the crown of the head, and fix them about the centre of the frontal bone, finish by tying the remaining ends of the frontal to the ends of the lower bandage, thus keeping the splint in a firm position.

In setting to work to repair a fracture you should be very careful to examine the mouth very carefully at first and to see that no teeth have been knocked out and have slipped down between the fractured parts, if such be the case the offending tooth should be removed. The next part of the operation is to take the model very carefully (in all cases be especially careful to handle your patient as gently as possible, as the pain attending a fractured jaw is very great), one of the best materials to take the model is with gutta-percha as it is much more springy and does not get so hard in the mouth as "Stent," and in removing it from the mouth it comes away much easier, and also the pain to the patient is much less.

A model of both the upper and under jaw should always be taken, and when you have the plaster cast ready, cut the model at the point of fracture, and as near as you can repair the model, making the bite articulate as near as possible with the model of the opposite jaw, a splint is made to hold the parts in position until they heal, there are three splints which are commonly used which I shall now describe, they are Gunning's, Kingsley's, and Hammond's splints.

The Gunning splint is practically two splints joined together, the way in which it is constructed is by making two ordinary splints and joining them together in the molar region, propping the bite sufficiently to allow a space to be left open in the front of the mouth, through which food may be given, and the mouth syringed out with disinfectants so as to keep it free from septic matter, which is an operation that should be well looked after, as the splint has to be worn for a considerable time, there should also be holes drilled in the vulcanite round about the necks of the teeth to allow any suppurating matter to exude through which may be washed away by syringing.

A modification of this splint can be used by simply making a splint for the fractured jaw and not capping the opposing teeth, but by letting them bite into blocks of vulcanite which prop the bite but do not cover up the teeth entirely, this is Mr. W. Hern's method, which he says answers very well and is not nearly so clumsy as the ordinary splint, therefore it can be put in and out of the mouth with comparative ease, whereas the ordinary splint is a rather large apparatus and is difficult to get in and out of the mouth.

Kingsley's splint is made by capping the teeth in the ordinary way, and fixing a piece of thick wire along the side of the splint from the sides of the molars and bicuspids out between the lips, and then bending it back along the cheek towards and on a level with the bottom of the ear, then by fixing a bandage to one wire and carrying it under the chin and catching it in the wire of the opposite side (having previously placed a pad of wool or lint under the chin to prevent galling), bringing it back again and catching it in the wire where the bandage commenced, having done this two or three times fix the bandages and the operation is finished.

The next splint that I shall describe is the Hammond splint and which is generally considered the best, it is made of wire which may be gold, silver, or tinned iron, the last of which is the preferable. When you have the model ready, bend the wire as near as possible to the arch of the teeth so as to make a loop of wire going all round the teeth, starting say from the buccal side of the molars and coming in front of the incisors and on towards the molars, being then brought right round the posterior wall of the last molar all along the inside of the teeth, to meet the wire at the starting point, the ends should be then soldered together and if any of the teeth are missing cross pieces from the outer to the inner wire should be soldered to strengthen the splint, the next thing to do is to place the splint into position, and by passing fine binding wire between the necks of the molar teeth catching the inside wire of the splint, then bringing the binding wire round the tooth the two ends should be twisted to hold the splint in position, these sutures are placed one on each of the first molars, first bicuspids and laterals, or any tooth that may seem fit; if any of these teeth be missing the jaw should be set; you next twist up the ends of the binding wires as tightly as possible and bend the ends of the wire along the gum margin so as not to irritate the cheek or lips.

These three splints are the general ones in use.

If at any time you are called upon to treat a fracture that has been neglected, and where a lot of the bone has been lost, the jaw become "V" shaped and the articulation practically gone, you should try to expand the jaw to its normal position and replace the lost bone and teeth by an artificial denture.

Such a case I saw treated about four years ago.

The patient was a laboring man, who, as he was driving fell out of the cart and fractured his lower jaw very badly, all the lower front teeth, excepting one of the canines, were either knocked out or had to be removed, the bone also necrosed and came away, he was treated in a general hospital for a few months and sent out as cured, having the bicuspid on one side of the mouth almost meeting the canine on the opposite side.

When I saw him he had practically no biting power whatever, and only lived on soft food, etc.

He was then placed under a dental surgeon, who taking a model, a vulcanite splint was made, at the same time capping the remaining teeth, the splint was then cut in two between the bicuspid on one side and the canine on the other; in this situation a wedge of compressed wood was fixed which gradually expanded the arch, the wood had to be removed every few days until there was room to get a jack-screw in position, a new case was then made with a jack-screw inserted and after a month or so the jaw was expanded sufficiently, after having worn the splint for some time and being able to masticate fairly well on it, he had an artificial denture made to fill up the space, and can now eat as well as if nothing had happened. I saw him a few months ago, and he told me that if he leaves the case out of the mouth for a few hours or a night, he has great difficulty in getting the case back again, as the union only being fibrous there is always a great tendency of the parts to close, and on one occasion he lost his plate for just over a day, and being unable by himself to place it in its proper position, applied to the dentist, who not without considerable trouble readjusted the splint.

In conclusion, I must thank you all very much for listening to my paper, hoping you will overlook any deficiencies on my part in attempting to write a paper on so large a subject. I have also to thank Mr. W. Hern very much for all the specimens he has been kind enough to lend me to pass round for your inspection.—*Dental Record.*

